# Efficacy of Biologics in the Management of Pediatric Asthma: A Single-Center Retrospective Study

**DOI:** 10.7759/cureus.101905

**Published:** 2026-01-20

**Authors:** Andy P Huang, Mahima Silas, Christie G Cherian, Floyd R Livingston

**Affiliations:** 1 Medicine, University of Central Florida College of Medicine, Orlando, USA; 2 Pulmonology, Nemours Children's Hospital, Orlando, USA

**Keywords:** asthma exacerbations, asthma management, biologic agents, biologic therapies, pediatric asthma, poor asthma outcomes, refractory asthma

## Abstract

Background

Biologics are monoclonal antibodies that target immune mediators of allergic asthma. Their use in children is not as well studied as in adults, and many indications are extrapolated from data conducted with adults. This study aims to evaluate the efficacy of biologics in improving respiratory function and reducing asthma-related morbidities in children and adolescents in a single health system, to compare efficacy between different biologics, and to explore whether differences in baseline biomarkers correlate with differing outcomes.

Methods

With IRB approval, a retrospective chart review study was conducted on patients with a diagnosis of asthma initiated on a biologic between 2019 and 2023. Indicators of morbidity (asthma severity, number of steroid courses, emergency room visits, hospitalizations), pulmonary function, and biomarker levels (IgE, eosinophils) were assessed for a year before and after biologic initiation. Outcomes pre- and post-biologic initiation were compared using dependent sample t-testing, conducted using IBM SPSS Statistics for Windows, Version 29 (Released 2023; IBM Corp., Armonk, New York, United States).

Results

One hundred and seventy-six subjects were included in our study. Biologics overall reduced morbidity and improved pulmonary function in children. Morbidity was decreased specifically with omalizumab and dupilumab, while pulmonary function also improved with dupilumab. Mepolizumab, benralizumab, and tezepelumab had small sample sizes, which limited the power of results from their groups. No significant differences in outcomes were noted between groups with different baseline biomarker levels.

Conclusions

We find that initiation of biologics in children and adolescents with poorly controlled asthma is associated with improvements in terms of morbidity and pulmonary function. Further data is needed on agents less commonly prescribed and for individual agents based on biomarker levels to better understand what role these agents have in managing pediatric asthma and help guide their continued use.

## Introduction

Control of severe asthma has long been challenging with current standard therapies of inhaled corticosteroids (ICS) and bronchodilators. The advent of monoclonal antibody-derived medications, termed biologic therapy, has revolutionized the treatment of severe asthma, with the introduction of the anti-IgE biologic omalizumab in 2003 and newer biologics targeting inflammatory mediators such as IL-4, IL-5, and IL-13 in later years. Dupilumab, omalizumab, and mepolizumab are approved by the Food and Drug Administration (FDA) for children aged six and up, while tezepelumab is approved for adolescents aged 12 and up [1]. Benralizumab's approval was expanded in April 2024 to children aged six and up, although approval was only for ages 12 and up during the period of our study. Despite these advances, the introduction of biologics has brought about questions regarding their use, such as their degree of benefit, how the efficacy of each biologic differs, and which biomarkers can be used to determine the drug of choice. 

In recent years, evidence has demonstrated the efficacy of certain agents like dupilumab in improving asthma control and quality of life with good tolerance in the pediatric population [2-4]. However, biologics in pediatric asthma are overall not as well studied compared to their use in adults, and many indications for biologic use in pediatrics are extrapolated from data conducted with adults [5]. Further studies are needed to better understand how they can be best used in this context. Current guidelines dictate that biologics are generally used for severe asthma refractory to non-biologic therapeutics and in patients who possess biomarkers indicative of T2 inflammation as indicated by elevated IgE, peripheral eosinophils, and fraction exhaled nitric oxide (FeNO) [1]. Peripheral eosinophil levels have been shown to be predictive of asthma exacerbations in children, while IgE-mediated allergen sensitization was found to be correlated with adverse outcomes as well [6,7]. Omalizumab in particular has been found to significantly reduce severe exacerbations in allergen-sensitizer asthmatic children [8]. Other biologics like mepolizumab and dupilumab have been found to produce similar reductions in adverse outcomes [1]. Data has also shown that biologics like dupilumab lead to significant improvements in forced expiratory volume in one second (FEV1%) [1]. 

Our study aims to assess differences in asthma-related morbidity and pulmonary function in pediatric patients initiated on biologics in a single health system, to compare these changes between different agents, and to explore whether patients with differences in baseline biomarkers (IgE, eosinophils) demonstrate differing outcomes.

Hypothesis

We hypothesize that pediatric asthma patients initiated on biologic therapy generally experience significantly less asthma exacerbations and associated emergency room (ER) visits, hospitalizations, and steroid courses after a year of therapy. We also predict that patients will overall demonstrate improved pulmonary function, with possible differences in response rate based on baseline respiratory status. The degree of improvement and differences between agents, however, may exhibit heterogeneity.

## Materials and methods

Data sourcing

Institutional review board approval was obtained from the Nemours Office of Human Subjects Protection (approval number: 2208163). Data was collected from the electronic medical record system of Nemours Children's Health system in Delaware and Florida. For our study cohort, patients who were initiated on biologics for the purpose of asthma control between 1/1/2019 and 1/1/2023, based on chart review, were identified and included in the study. Subjects initiated on biologics for alternative diagnoses such as eosinophilic esophagitis or atopic dermatitis, without a diagnosis of asthma, were excluded. Subjects who were lost to follow-up or otherwise lacked records for one year before and after biologic initiation were also excluded. The use of concomitant therapies for asthma was not taken into consideration.

Data collection

Baseline characteristics for each subject were assessed for a one-year interval prior to the first injection of biologic. Characteristics assessed were number of asthma exacerbations, number of steroid courses prescribed for asthma, number of pulmonary-related ER visits, number of pulmonary-related hospitalizations, and average pulmonary function.

Asthma exacerbations were defined as any encounters with ICD-10 codes associated with asthma exacerbation (J45.21, J45.31, J45.41, J45.51, J45.901) or encounters with oral corticosteroids prescribed for asthma. Steroid courses were defined as any individual instance where a course of oral corticosteroids was prescribed for asthma. ER visits were defined as any encounter in the ER setting with asthma exacerbation as the primary diagnosis, with or without subsequent admission. Hospitalizations were defined as any admission to the inpatient wards or intensive care units for asthma exacerbation or respiratory failure. Encounters (including but not limited to in-person appointments, telehealth appointments, telephone encounters, and medication refills) for one year prior to the subject's first biologic dose were analyzed for occurrences of any of the four outcomes, with means for each outcome being calculated.

Pulmonary function was assessed based on subjects' average percent predicted pre-bronchodilator FEV1% and percent predicted pre-bronchodilator forced expiratory flow at 25-75% of forced vital capacity (FEF25-75%) over the one-year interval. All FEV1% and FEF25-75% results from pulmonary function testing from one year prior to biologic initiation were recorded and their mean calculated.

Results from biomarker testing, including IgE count, absolute eosinophil count, and FeNO, were also collected during this interval and were recorded as averages over the one-year period if tested multiple times, with all values included if present. 

The same characteristics were assessed for the one-year interval following the initiation of biologic. Subjects who failed to complete at least one year of therapy were included and analyzed as intention-to-treat. Baseline demographic information and asthma severity were also recorded for the visit where biologic was initiated. All collected data were stored using the REDCap software hosted by Nemours Children's Health [9,10].

Statistical analysis

Dependent sample t-testing was used to compare differences in outcomes and pulmonary function and to calculate 95% confidence intervals. Two-tailed testing was utilized. This was chosen to allow comparisons of pre- and post-biologic initiation means for each outcome analyzed both for the aggregate sample and for individual biologics. Dependent sample testing was specifically chosen because pre- and post-biologic results were measured for the same subjects. Statistical analysis was conducted using IBM SPSS Statistics for Windows, Version 29 (Released 2023; IBM Corp., Armonk, New York, United States).

## Results

We analyzed data from 176 initiations of biologic agents for asthma control across our health system (Table 1). As expected, most biologics were started for severe persistent asthma, with dupilumab being the most popular agent followed by omalizumab. 

**Table 1:** Study sample characteristics Highlight of the baseline characteristics of subjects analyzed in this study. Categorical variables are displayed as N (%). Continuous variables are displayed as mean±standard deviation. FEV1%: forced expiratory volume in one second; FEF25-75%: forced expiratory flow at 25-75% of forced vital capacity

Characteristic	Value
# of subjects	176
Subject age (years)	12.49±3.40
Male sex	92 (52.3%)
Female sex	84 (47.7%)
Black/African American ethnicity	72 (40.9%)
White/Caucasian ethnicity	52 (29.5%)
Hispanic/Latino ethnicity	38 (21.6%)
Asian ethnicity	1 (0.6%)
Other ethnicity	13 (7.4%)
Severe persistent asthma	127 (72.2%)
Moderate persistent asthma	46 (26.1%)
Pre-biologic FEV1%	83.08%±19.37%
Pre-biologic FEF25-75%	63.36%±28.40%
IgE (IU/mL)	1190±1883
Eosinophils (cells/mm^3^)	573±868
Subjects undergoing allergy testing	103 (58.5%)
Dupilumab initiated	65 (36.9%)
Omalizumab initiated	56 (31.8%)
Benralizumab initiated	23 (13.1%)
Mepolizumab initiated	21 (11.9%)
Tezepelumab initiated	11 (6.3%)

**Figure 1:**
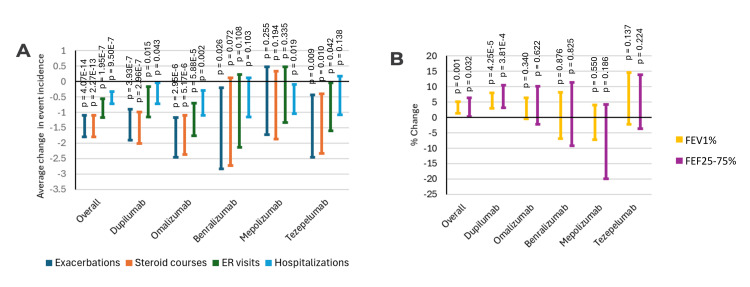
Change in asthma clinical status following biologic initiation (A) Average changes in the incidence of asthma-associated morbidities between one year before and after biologic initiation. (B) Average change in pulmonary function between one year before and after biologic initiation. All intervals indicate 95% confidence intervals. P-values shown are derived from dependent sample t-testing. FEV1%: forced expiratory volume in one second; FEF25-75%: forced expiratory flow at 25-75% of forced vital capacity; ER: emergency room

Subjects overall demonstrated significant reductions in all four pulmonary-related morbidities studied following the initiation of biologic therapy (Figure 1A). All four morbidities were also significantly reduced in subjects started on the two most commonly prescribed biologics at our institution: dupilumab and omalizumab. However, results were mixed for the other biologics, with significant reductions in some but not all studied morbidities. Pulmonary function, as measured with FEV1% and FEF25-75%, also demonstrated significant improvement overall in patients started on biologics and those started on dupilumab (Figure 1B). Improvements in pulmonary function for those initiated on other agents, however, were not found to be significant. 

**Figure 2:**
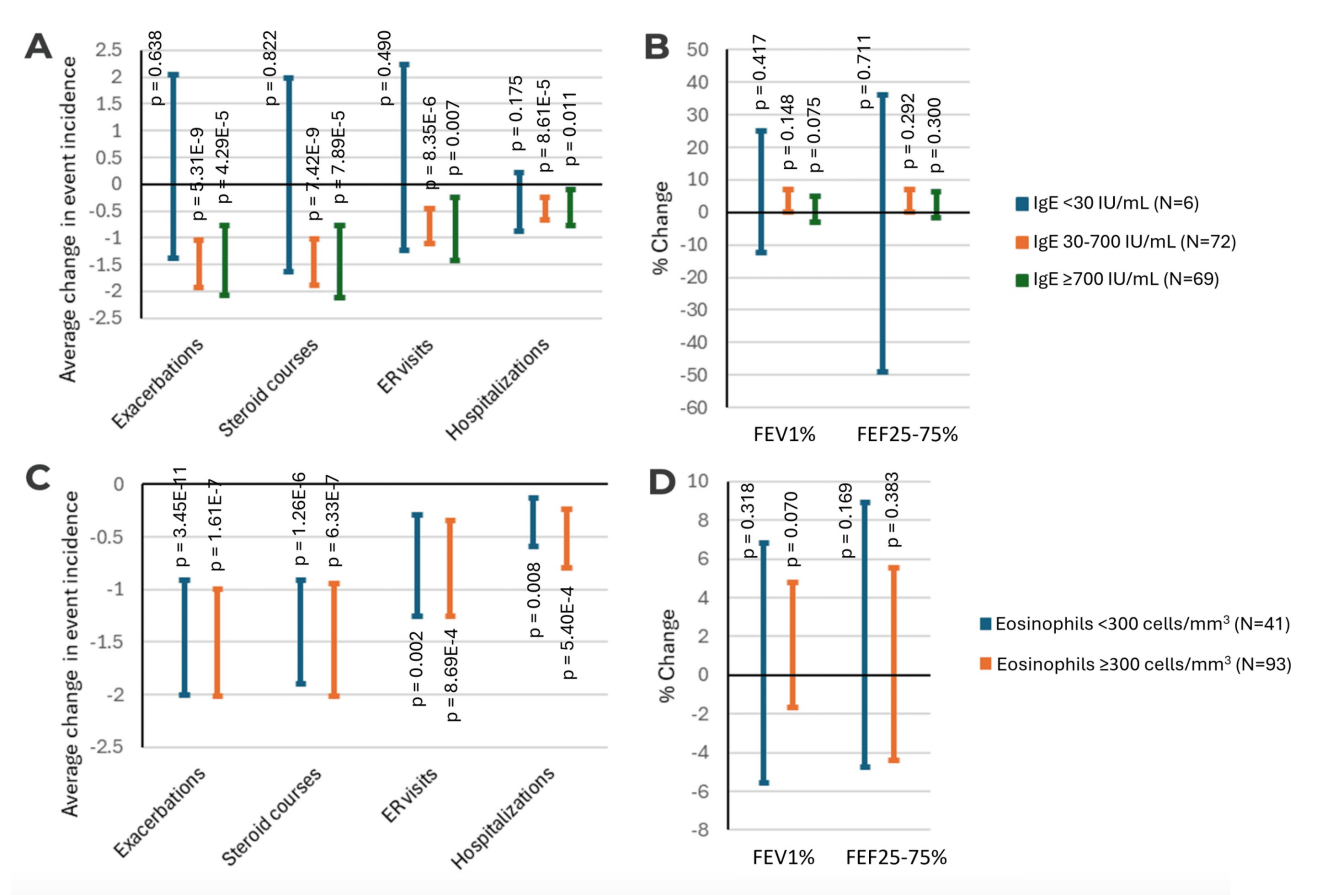
Relationship between asthma biomarkers and outcomes (A) Changes in the incidence of asthma-associated morbidities stratified by baseline IgE. (B) Average change in pulmonary function stratified by baseline IgE. (C) Changes in the incidence of asthma-associated morbidities stratified by eosinophil count. (D) Average change in pulmonary function stratified by eosinophil count. P-values shown are derived from dependent sample t-testing. FEV1%: forced expiratory volume in one second; FEF25-75%: forced expiratory flow at 25-75% of forced vital capacity; ER: emergency room

We further stratified our findings based on each subject's baseline IgE and eosinophil count to determine if reductions in morbidity and improvements in pulmonary function differ based on baseline biomarkers. A cutoff of 30 for IgE was selected as this is used as the minimum initiation cutoff for omalizumab, while a cutoff of 300 was used for eosinophils as this is the minimum cutoff for mepolizumab and benralizumab. FeNO counts were not used for analysis as it was not commonly used at our institution. Overall, we found that subjects with IgE counts of 30-700 and greater than 700 showed no significant differences in morbidity reduction and pulmonary function improvement compared to each other (Figure 2A-2B). Very few subjects had a baseline IgE lower than 30. Similarly, subjects with absolute eosinophil counts of less than or greater than 300 showed no significant differences in outcomes (Figure 2C-2D). 

## Discussion

Omalizumab has been shown to significantly reduce asthma exacerbation incidence and steroid doses required for exacerbations in randomized controlled trials and specifically in the pediatric population [8,11]. Dupilumab has similarly been shown to be effective in reducing exacerbations in children and adolescents [12,13]. Mepolizumab, benralizumab, and tezepelumab have been similarly demonstrated to be effective at reducing exacerbations in the general population [14-17]. Our results showed modest reductions in occurrences for exacerbations, steroid courses, ER visits, and hospitalizations for these agents, though not all findings reached statistical significance. We believe that this is most likely due to small sample sizes for these groups, as these three agents were less commonly used. Another possibility is low baseline occurrences of each outcome prior to biologic initiation, resulting in less room for improvement. However, based on the overall observed trends for biologics in our study, we believe children with severe and uncontrolled asthma overall benefit from biologic therapy.

Current evidence suggests that biologics typically cause modest but significant improvements in pulmonary function. The biologic with the strongest evidence for pulmonary function improvement is dupilumab, which has been specifically shown to induce statistically significant improvements in FEV1% in children in clinical trials [12,13]. Evidence for omalizumab is less definitive, with no significant changes in FEV1% and FEF25-75% noted for children in 2001 [11]. Our results appear to be consistent with these findings, with significant improvements in FEV1% and FEF25-75% with dupilumab and nonsignificant improvements with omalizumab. No significant changes in pulmonary function were observed for other agents, which we again attribute most likely to small sample sizes and baseline pulmonary function that is within normal limits for many subjects. Benralizumab and tezepelumab in particular have been shown to be effective at increasing FEV1% in the past [15,17]. Assessment of biomarkers, such as IgE count and eosinophil count, has been an important prerequisite for biologic initiation given their mechanisms of action. We selected IgE cutoffs of 30 and 700 and an eosinophil cutoff of 700 for our study as these are cutoffs used for determining eligibility for omalizumab, dupilumab, and benralizumab [1]. Our results indicate that subjects with IgE counts of 30-700 and >700 showed similar findings with regard to morbidity and pulmonary function with biologics overall. Likewise, subjects with absolute eosinophil counts of greater or less than 300 demonstrated similar outcomes. Very few subjects with IgE <30 were initiated on biologics, so no meaningful conclusions could be drawn for this group. Overall, our findings raise the possibility that biologics may benefit some children with uncontrolled asthma even in the absence of elevated IgE or eosinophil counts.

Our study has several limitations. Our single-center retrospective study design limits the scope of our findings and the ability to draw definitive conclusions about the use of biologics in general. Because of the retrospective nature of this study, we relied on individual clinician-assigned ICD codes in the medical record to determine asthma severity, which we recognize can be variable and thus limit the reproducibility of our findings. Classification of asthma severity based on established criteria in a prospective format would improve replicability. A prospective format would also allow for the inclusion of other measures, such as asthma control scores and measures on quality of life, which are not routinely collected in clinical practice but are important in measuring the impact of interventions on asthma.

We believe that larger sample sizes for individual agents would help assess the degree of benefit derived from them. Comparisons for mepolizumab, benralizumab, and tezepelumab would particularly benefit from this. Furthermore, larger sample sizes that support analysis of biomarker subgroups for individual agents would be necessary to better understand their impact given the differing mechanisms of action and initiation criteria. As our biomarker analysis is limited to only biologics in general, we cannot draw conclusions about individual agents. A larger sample size would also allow for the controlling of additional confounding factors that may influence our results, such as previous compliance with first-line therapies, comorbid atopic disease, sociodemographic factors, adherence to biologics during the study period, and others. We also did not include any matched control groups of subjects who were never initiated on biologics, which may introduce bias relating to natural disease course variation, other changes in background care over the study period, or regression to the mean. Overall, we believe that a prospective larger randomized controlled study with non-biologic therapy controls would be indicated to better define the biological therapy effects on pediatric asthma outcomes.

## Conclusions

Overall, we find that biologic initiation is associated with improvements in morbidity and pulmonary function in children and adolescents with asthma poorly controlled with conventional therapy. However, further data is needed on agents less commonly prescribed, which will help better determine what role these agents have in managing pediatric asthma. Further studies are also needed regarding how response varies based on baseline biomarker levels, as this can improve understanding on when certain agents may be more effective than others. A better understanding of how pediatric asthma patients benefit from biologics overall will help guide their continued use in this group.
